# Ensuring the Safe Use of Bee Products: A Review of Allergic Risks and Management

**DOI:** 10.3390/ijms262412074

**Published:** 2025-12-15

**Authors:** Eliza Matuszewska-Mach, Paulina Borysewicz, Jan Królak, Magdalena Juzwa-Sobieraj, Jan Matysiak

**Affiliations:** 1 Poznan University of Medical Sciences, Department of Inorganic and Analytical Chemistry, Rokietnicka 3 Street, 60-806 Poznan, Poland; 2 Poznan University of Medical Sciences, The Students Scientific Society, Rokietnicka 5 Street, 60-806 Poznan, Poland; 3 Poznan University of Medical Sciences, Department of Hypertensiology, Angiology and Internal Medicine, Długa 1/2 Street, 61-848 Poznan, Poland; 4 Poznan University of Medical Sciences, Department of Pediatric Anesthesiology and Intensive Therapy, Carol Jonscher’s Clinical Hospital, Szpitalna 27/33 Street, 60-572 Poznan, Poland

**Keywords:** allergens, anaphylaxis, food hypersensitivity, honeybee products

## Abstract

Honeybee products (HBPs), including honey, bee pollen, bee bread, royal jelly, propolis, beeswax, and bee brood, are increasingly used in food, nutraceutical, and cosmetic contexts. Because of their natural origin, HBPs can provoke allergic reactions ranging from localised dermatitis to life-threatening, systemic anaphylaxis. As the use of bee products for health purposes grows in apitherapy (a branch of alternative medicine), raising public awareness of their potential risks is essential. This narrative review synthesises the clinical manifestations of HBP allergy, culprit allergens present in each product, immunological mechanisms, diagnostic approaches, at-risk populations, and knowledge gaps. The analysis of the available literature suggests that, although relatively rarely, HPB may trigger allergic reactions, including anaphylactic shock. The sensitisation mechanism may be associated with both primary sensitisation and cross-reactivity and can be classified into type I (IgE-mediated) and type IV (T-cell-mediated). However, bee bread appears less allergenic than other HBPs, potentially due to lactic fermentation that can degrade allergenic proteins. Severe reactions following intake of bee bread have not been reported to date. Management of HBP allergic reactions centres on avoiding the products, educating about the risks, and providing more precise product labelling, specifying the allergen content. Individuals with atopy and beekeepers are at heightened risk of developing anaphylaxis; therefore, they should be particularly aware of the potential dangerous consequences of HPB use. Further research is needed to clarify the mechanisms of HBP allergies and improve safety for all users.

## 1. Introduction

Honeybee products (HBPs), including honey, bee pollen, bee bread, royal jelly, propolis, beeswax, and bee brood, are increasingly utilised in various industries, such as food, nutraceuticals, and cosmetics [1,2]. They exhibit a wide range of health-promoting properties, making them valuable in the branch of alternative medicine known as apitherapy [3]. This natural method of treatment is gaining popularity and, because of growing health consciousness, HBPs are frequently used by people caring for their well-being—but often without medical consultation. The benefits of using HBPs are well-documented, and their effects on the human organism stem from their complex composition [4]. However, this complexity can also be the reason for adverse reactions, with allergies being the most common and dangerous.

Our modern lifestyle exposes us to highly processed food and artificial additives, which can harm our health. These factors can contribute to the development of obesity, metabolic disorders, depression, and a shorter lifespan [5]. Using synthetic, chemical-based cosmetics also carries the risk of side effects, such as skin sensitivity and allergies, and may even increase the chance of developing skin cancer and other tumours [6]. Therefore, current trends indicate a strong preference for natural products over processed or synthetic alternatives, driven by the widespread perception that “natural” is synonymous with “healthy”. However, it is essential to remember that “natural” does not always mean “safe” [7]. While bee products are generally considered safe, they can occasionally cause allergic reactions in sensitive individuals, including potentially life-threatening anaphylaxis [8]. Raising consumer awareness of the dangers associated with natural products, such as HBPs, is essential to prevent adverse health outcomes (also underdiagnosed), particularly in children.

While numerous recent reviews have extensively documented the nutritional composition and therapeutic applications of HBPs [4,9,10,11], there is a paucity of comprehensive literature explicitly dedicated to their safety profile. Most existing surveys focus individually on major products, such as honey or bee pollen, often overlooking the risks associated with emerging novel foods from the hive. This review distinguishes itself by providing a holistic safety assessment of the entire hive spectrum, including less frequently discussed products such as bee bread, beeswax, and bee brood. Furthermore, unlike reviews that focus solely on clinical case reports, we integrate clinical data with toxicological in vitro findings and provide a comparative analysis of raw bee pollen versus fermented bee bread to highlight the impact of processing on allergenicity. Consequently, this review summarises adverse reactions, focusing on allergic responses, following the consumption and topical usage of honeybee products: honey, bee pollen, bee bread, royal jelly, propolis, beeswax, and bee brood. Honeybee venom (HBV) is not included, as it is not typically used as an oral dietary supplement.

The specific aims of this review include: (a) to describe the clinical spectrum of allergic reactions for each product, with particular attention to anaphylaxis; (b) to identify the primary allergens in HBPs; (c) to discuss the mechanisms of sensitisation, including cross-reactivity; (d) to summarise diagnostic and management approaches.

To achieve these aims, a literature search was conducted using the Web of Science and Google Scholar databases. While the review covers relevant data starting from 1950, the majority of the included studies were published after 2010. The search strategy utilised the following keywords: honeybee products, honey, bee pollen, bee bread, royal jelly, propolis, beeswax, bee brood, allergy, allergic reaction, anaphylaxis, allergenicity, immunology, sensitisation, case report, in vitro models, and in vivo models.

## 2. Immunological Mechanisms of Sensitisation to HBPs

Allergy can occur through both primary sensitisation and cross-reactivity. Primary sensitisation refers to the development of immunoglobulin E (IgE) production that happens right after an allergen contacts either the gastrointestinal tract, the respiratory tract, or the skin [12]. It is a direct sensitisation to a protein unique to a bee product, e.g., Major Royal Jelly Proteins. On the other hand, cross-reactivity refers to already-existing sensitisation to an external allergen that is also present in the bee product. In other words, cross-reactivity occurs when allergens share the same epitopes, which are short peptides present on the surface of antigens that are recognised by adaptive immune responses [13,14,15]. This is the most common mechanism for honey and bee pollen allergy, called pollen food allergy syndrome (PFAS).

The term “hypersensitivity” was first introduced in 1951, and in 1963, it was defined as: “an undesirable, uncomfortable or damaging response that arises from an overreaction of the adaptive immune response” [16]. The Gell and Coombs hypersensitivity reactions are classified into four distinct types: type I—immediate, IgE-mediated; type II—cytotoxic, antibody, and Fc-receptor-mediated; type III—immune-complex-mediated; and lastly, type IV—delayed-type, T-cell-mediated [16,17]. In terms of HBP allergy potential, hypersensitivity reactions can be classified into type I (IgE-mediated) and type IV (T-cell-mediated). Type I reactions are immediate reactions, including urticaria, angioedema, asthma, and anaphylaxis. They can be observed after contact with honey, royal jelly, propolis, and bee pollen.

Before proceeding to the discussion of the type I hypersensitivity reaction, it is important to provide a brief overview of IgE—as it is a pivotal molecule taking part in this type of reaction. Its size is equal to around 190 kDa, and in terms of structure, it resembles other immunoglobulins, as it is also a tetramer and contains two heavy and two light chains. In terms of pharmacokinetics, IgE has a short half-life—of around one day (but when bounded to mast cells (MCs), it can increase to a few weeks), and its serum concentration is rather low—it reaches approximately 50 ng/mL because, in part, it is continuously removed and broken up by endosomes [18,19,20]. It is also important to note that elevated levels of IgE are present among patients suffering from asthma, atopic dermatitis, or allergic rhinitis [20]. In type I hypersensitivity, IgE is first introduced during the sensitization phase. The type I reaction is described in more detail below. Nonetheless, the first part of this reaction—the sensitization phase—is when allergen-specific IgE (sIgE) is produced. This happens after the first encounter with the allergen; the antigen-presenting cells (APC) start to stimulate differentiation of naïve T cells into Th2 cells, which then secrete cytokines (interleukin-4 (IL-4) and interleukin-13 (IL-13)). Under the influence of these cytokines, B cells undergo class switching and sIgE is produced. This IgE class switching can either happen locally—in the nasal, bronchial mucosa—or in the lymphatic tissues, which are adjacent to where the contact with allergen took place. However, as stated in an article by Shamji et al., the exact place of class switching is not known yet [21]. MCs and basophils (BAS) have high-affinity FcεRI receptors on their surface [22], which the produced sIgE is then able to bind to, and therefore the molecule becomes “coated” (or in other words, “sensitised”) with IgE. This step provides a quick reaction after subsequent exposure to the allergen. Then, with another exposure to the allergen, it binds and cross-links with IgE on the surface of the MCs, and therefore triggers a quick immunological response. With that comes the degranulation of the MCs, which causes the release of preformed mediators (e.g., histamine, tryptase, and other enzymes), followed by the synthesis and release of lipid mediators (e.g., leukotrienes and prostaglandins) [18,23]. These substances are responsible for the clinical manifestation of symptoms of allergy—vasodilation, smooth muscle cell contraction, and mucus secretion. Depending on the extent and site of the release of these mediators [24], what is seen clinically starts from urticaria, conjunctivitis, or allergic rhinitins, and can end as a systemic response like anaphylaxis.

**Figure 1:**
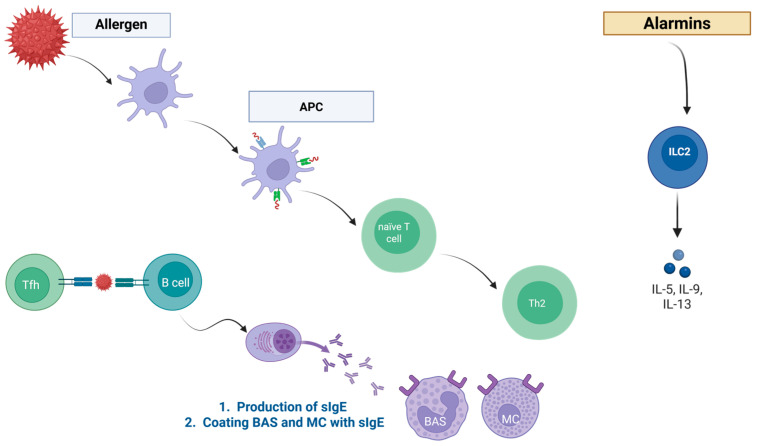
Sensitisation phase in a type I reaction. Created in BioRender. Matysiak, J. (2025) https://BioRender.com/m0zqiwf.

**Figure 2:**
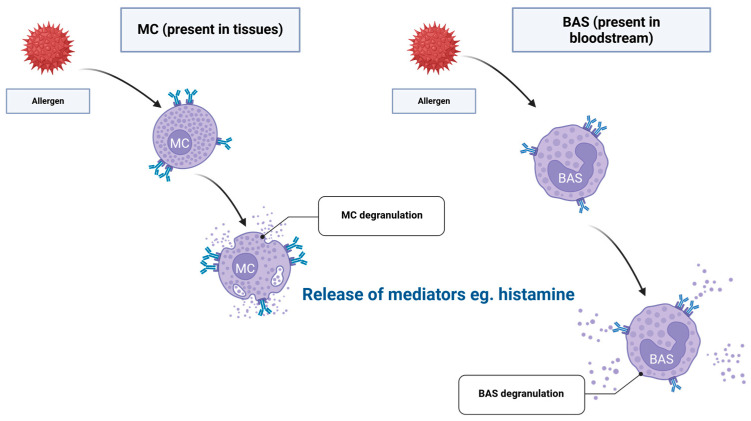
Effector phase in type I reaction. Created in BioRender. Matysiak, J. (2025) https://BioRender.com/6yg684z.

The mechanism of the type I reaction is divided into two phases: the sensitisation phase and the effector phase, as pictured in Figure 1 and Figure 2, respectively. The sensitisation phase occurs after the initial encounter with an allergen—here, the antigen-presenting cells present the antigen to the naïve T-helper cells (Th). APCs can either be dendritic cells (DC), B lymphocytes (B), or macrophages (Mφ). Before presenting to naïve T cells, APCs process and display the allergen-derived peptide fragments on their surface in association with major histocompatibility complex class II (MHC II) molecules for recognition by naïve T cells. Surface molecules of the APCs, and their secreted metabolites and cytokines, are allowing for the activation and differentiation of naïve T cells into different immune cell subtypes: Th1, Th2, Th17, Tc1, Tc2, and Tc17, as well as regulatory T cells (Treg). Afterwards, innate lymphoid type 2 cells (ILC2) are activated by the cytokines released by alarmins, which are a type of epithelial cells. These cytokines include IL-25, IL-33, and thymic stromal lymphopoietin (TSLP). Once activated, they can release high levels of type 2 cytokines (e.g., interleukin 5 (IL-5), IL-13, and interleukin 9 (IL-9)), thereby amplifying the type 2 immune response, recruitment of eosinophils, and production of mucus. This cascade drives the differentiation of naïve T cells into Th2 and Tc2 subsets. IL-4 and IL-13 facilitate immunoglobulin class-switch recombination in B cells, which leads to the generation of allergen-specific IgE (sIgE), and then also enhances the recruitment of Th2 cells into affected tissues. T follicular helper (Tfh) cells—specialised CD4^+^ T helper cells—are essential for orchestrating B-cell maturation and the formation of high-affinity antibody responses. Within germinal centres, Tfh cells deliver key signals to B cells, including cytokines such as IL-4 and IL-21 and costimulatory interactions such as CD40L (the cluster of differentiation 40 ligand), thereby promoting class-switch recombination and subsequent IgE production. MCs and BAS, which express the high-affinity IgE receptor FcεRI, recognise Fc part of IgE. sIgE binds with high affinity and irreversibly with FcεRI—making it sensitised to subsequent exposure to a given allergen. This marks the end of the sensitisation phase [16].

Then, the effector phase takes place after another contact with the same allergen. There, the allergen crosslinks IgE bound to the surface of MCs and BAS, leading to their degranulation. MCs are distributed throughout various body tissues, while BAS circulate in the bloodstream. The release of preformed mediators—such as histamine, but also heparin, proteases, cytokines, prostaglandins, and leukotrienes—from these cells into the surrounding tissue environment results in clinical symptoms, including vasodilation, bronchoconstriction, and enhanced mucus production [16].

**Figure 3:**
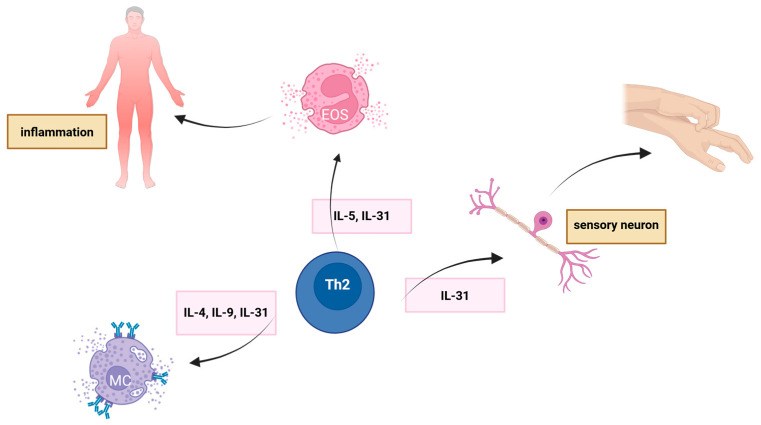
The effects of Th2 cell activation. Created in BioRender. Matysiak, J. (2025) https://BioRender.com/ijw7vb5.

Type IV, and more precisely, type IVb reactions cause delayed reactions like allergic contact dermatitis, as seen in reactions after contact with propolis. Here, the core drivers are Th2 cells, ILC2, NK-T (natural killer T) cells, eosinophils, and Mφ. Th2 cells, when activated, release cytokines: interleukin 4 (IL-4), interleukin 13 (IL-13), interleukin 5 (IL-5), interleukin 9 (IL-9), interleukin-31 (IL-31), and eotaxins I-III, in order to activate eosinophils and MCs [16,25]. This is shown in Figure 3. IL-4 and IL-13 promote IgE class switching in B cells, and IL-5 recruits eosinophils, contributing to inflammation and tissue damage; then, in turn, through degranulation, eosinophils release endogenous proteases into their surroundings, which further causes tissue injury and damage as well as barrier disruption. Sensory neurons are activated by IL-31 (which, for the main part, is produced by the aforementioned Th2 cells, but it can also be produced by DCs and Mφ), which is the driving force for itching and neurogenic inflammation; hence its localization on sensory neurons as well as epithelial cells and keratinocytes. Th9 cells, induced by IL-4 and tumour necrosis factor beta (TNF-β), enhance sIgE production and MC growth. The response is amplified by ILC2, MCs, and alternatively activated macrophages. ILC2, Th2 cells, and dendritic cells (DC), when stimulated by IL-25, IL-33, and thymic stromal lymphopoietin (TSLP), coordinate the release of cytokines, disrupt epithelial barriers, and recruit eosinophils and BAS, which sustains chronic inflammation [16].

## 3. Allergenic Profiles of the Selected HBPs

### 3.1. Honey

Honey is a natural product produced by honeybees. Honeybees first collect nectar, honeydew, or resin, which they carry in their honey sacs and then store in honeycombs. There, it is mixed with the bees’ enzymes, then beekeepers wait until the honey matures before it can be harvested [26,27]. Around 300 different types of honey are available, and such a high number results from the various kinds of nectar that honeybees can collect [28]. The main components of honey are carbohydrates (fructose being the most abundant), but there are also proteins, vitamins (e.g., folate and vitamin C), amino acids, minerals (e.g., potassium being the most abundant), organic acids, aromatic compounds, and polyphenols [28,29]. Thanks to its chemical composition, honey possesses anti-bacterial, anti-viral, anti-parasitic, anti-tumour, and anti-inflammatory effects [30]. However, these health-promoting effects can be achieved after consuming around 50 to 80 g per serving, which is a relatively large amount [30]. Allergies to honey are rare, and if they happen, they are often connected to sensitisation to pollen from the *Asteraceae* family or bee-derived proteins [31,32,33], like Major Royal Jelly Protein 1 (MRJP1) [34].

Several cases of honey-induced allergic reactions are reported, both in children and adults [35]. Although rare, anaphylaxis can happen after honey consumption [31,32,33,36]. In one case published in 2025, a 65-year-old male undergoing mitomycin C treatment for urothelial carcinoma had recurrent grade III anaphylaxis of unknown aetiology [32]. In the two reported hospital visits, the patient had wheat bread, strawberry jam, and blossom honey for breakfast. The symptoms that he experienced included: erythema, collapse, and dyspnoea during one episode, and generalised flushing, hypotension, dyspnoea, tachypnea, defecation, and tongue swelling during another episode. Both of them required intensive care unit (ICU) stabilisation. Moreover, this patient had symptoms of rhinoconjunctivitis during spring and summer, and later assessment of food allergies showed this patient’s sensitivity to honey. After a 2-year follow-up, with total elimination of honey from the diet, the patient reported no anaphylactic reactions. Another case of anaphylaxis was seen in a 40-year-old woman who had first experienced generalised urticaria after consuming foods with honey, and then, secondly, experienced swollen lips, urticaria, and angioedema after accidental contact with a teaspoon with residual amounts of honey [33]. She did not have any history of atopic diseases nor sensitivity to *Hymenoptera*. There was also one case of anaphylaxis reported in Iraq, where a 35-year-old male developed symptoms ranging from mild to severe, including skin irritation, abdominal pain, vomiting, diarrhoea, dizziness, and fainting [36]. The symptoms occurred within 15 min after honey consumption. Another case concerns anaphylaxis in a 5-year-old boy suffering from allergic rhinitis and sensitivity to dust mites and *Plantago lanceolata* [31], which comes from the *Asteraceae* family. The patient’s family history stated that his father suffered from seasonal allergic rhinitis. The child was admitted to the hospital after experiencing generalised urticaria and breathing impairment, which happened unexpectedly after eating a meal of salmon and artisanal honey.

Interestingly, some studies have shown remarkable improvements in certain allergic illnesses, supporting the idea that honey may be an effective anti-allergic agent [37]. For example, a randomised controlled trial by Saarinen et al. [38] investigated this concept by having patients with birch pollen allergy consume either regular honey (RH) or birch pollen honey (BPH) before the allergy season, and comparing them to a control group using conventional medication. The results were significant: compared to the control group, patients using BPH experienced a 60% lower total symptom score and twice as many asymptomatic days, and used 50% less antihistamine medication. According to the results, pre-seasonal use of BPH could serve as a complementary therapy for birch pollen allergy. However, more research is needed to confirm this concept and to identify the specific bioactive phytochemical compounds responsible for the anti-allergic effects of particular types of honey.

In a study by Bauer et al. [39], the researchers investigated allergenic components in honey in vitro using sera obtained from patients. Patients were divided into three groups: group 1 consisted of 23 people allergic to honey (13–67 years old), group 2 consisted of 10 patients allergic to bee venom (6–67 years old), and the control group contained three people without allergies. The study was conducted using immunoblotting and IgE inhibition studies in relation to cross-reacting proteins between honey and bee venom. For this, the researchers first performed the characterisation of four different types of honey: forest, locust, chestnut, and sunflower. From that, sunflower (*Helianthus annus*, from the *Asteraceae* family) honey was chosen for further investigation. The result was that honey-allergic patients’ sera showed binding of IgE to the following strips of sunflower honey extract at the following molecular masses: 54 kDa, 60 kDa, 72 kDa, or to the double band at the molecular mass of 30 kDa (30 kDa, 33 kDa). The bands from the masses (54 kDa, 60 kDa, and 72 kDa) were also observed in the other kinds of honey tested, indicating that they could actually be of bee origin and not necessarily pollen proteins. The double band at 30 kDa was sunflower honey-specific, which here could, in fact, point to sensitization due to the aforementioned *Asteraceae* family pollen. Similarly, in an in vitro study by Burzyńska et al. [40], where researchers tested the allergenic properties of Polish nectar honeys, patients’ IgE showed binding with protein bands at the molecular weight of around 60 kDa.

Helbling and colleagues [41] have performed a series of both in vitro and in vivo tests in order to identify allergenic components of honey. The types of honey taken under investigation were rape, dandelion, and forest honey. There were 22 participants included in the study within the age range of 16 to 78 years old, and they all presented with a history of systemic allergic reactions after ingestion of honey. For in vivo tests, the skin-prick test (SPT) and scratch test were performed. The results were that 17 patients had a positive reaction to dandelion honey, and 10 each to rape and forest honey. Moreover, when tested for different pollen allergens, patients showing sensitization to dandelion also reacted to artemisia pollen—both from the *Asteraceae* family. Radioallergosorbent testing (RAST) was utilised for the in vitro part of the study. Here, it turned out that more than 11 patients showed reactions to artemisia and dandelion pollen [41]. This proves that the allergenicity of honey comes from the plant pollen present in this type of honeybee product.

On the other hand, there are pre-clinical in vitro studies about honey’s impact on the immune status of Ehrlich ascites tumours in mice. A study showed that pre-treatment with Egyptian honey inhibited tumour growth and enhanced the function of macrophages, B cells, and T cells [42,43]. In a study from 2013 [44], the authors have used an aqueous extract from fir honeydew honey. Using HaCaT cells, they showed that this aqueous extract was able to inhibit and reduce the production of tumour necrosis factor alpha (TNF-α) that induces matrix metalloproteinase 9 (MMP-9) [45]. The study was conducted in terms of wound healing, but it shows that components derived from this honey exhibit anti-inflammatory properties.

Honey contains allergens of both bee and plant origin. Known honey allergens of bee origin include Major Royal Jelly Protein 1 (MRJP1), which is the dominant protein in both royal jelly and honey. Interestingly, although MRJP1 is a recognised allergen, it may also be responsible for honey’s beneficial bioactivity. Specifically, MRJP1 has been linked to honey’s immunomodulatory effects and hepatocyte proliferation, while peptides derived from this protein exhibit potent antihypertensive and antimicrobial properties [46]. Moreover, although less abundant than MRJP1, several honeybee venom (HBV) proteins have been identified in various types of honey, including phospholipase A2 (Api m 1), hyaluronidase (Api m 2), acid phosphatase (Api m 3), melittin (Api m 4), and icarapin (Api m 10), which could potentially cause sensitisation. Other allergens include Api m 7 (a 39 kDa protein with protease activity), Major Royal Jelly Protein 8 (MRJP8) (Api m 11.0101a), Major Royal Jelly Protein 9 (MRJP9) (Api m 11.0201a), and, rarely, vitellogenin (Api m 12) [47].

Plant-derived allergens in honey depend on the botanical source from which the honeybees collected nectar and pollen [40]. Since bees visit hundreds of various flowers, the honey they produce naturally contains trace amounts of plant pollen. Thus, the resulting sensitisation to honey is often linked to an existing allergy to airborne pollen. The allergenic potential, therefore, depends on the specific seasonal plants available at the time of collection. Sensitisation is usually connected to pollen from the large *Asteraceae* (*Compositae*) family [39]. This family includes well-known allergenic factors such as dandelion, mugwort, goldenrod, sunflower, and artemisia. However, the allergenicity of honey is highly variable, influenced by factors such as geographic origin, climate conditions, and soil type. It is essential to note that individuals allergic to airborne pollen may experience systemic reactions upon consuming honey containing the same pollen allergens.

### 3.2. Bee Pollen

Bee pollen (BP) is a mixture of flower pollen collected by foraging bees, small amounts of nectar, and bees’ secretions. It shows various health-beneficial properties, such as antimicrobial, anti-fungal, anti-viral, anti-inflammatory, immunostimulating, hepatoprotective, anti-osteoporosis, anti-anaemia, and anti-cancer effects [48,49]. The recommended therapeutic dosage for adults is 20–40 g of pollen, taken three times a day before meals for one to three months. This treatment can be repeated twice to four times yearly [50]. The positive effects of BP are well-documented. However, although relatively rare, allergic reactions to ingested BP have also been reported. Paradoxically, this product is also believed to possess anti-allergy properties [49,51].

Several studies have reported the development of allergic reactions caused by BP in adults and children [35]. The most serious and life-threatening documented consequence of ingesting bee pollen was anaphylactic shock. Fortunately, dangerous BP-induced anaphylaxis is relatively rare. In one case, in a 40-year-old male, the first systemic allergy symptoms appeared one hour after BP intake [52]. This patient had a history of seasonal allergic rhinitis but no known sensitivities to foods, drugs, or *Hymenoptera*. Another case of anaphylaxis was observed in a 30-year-old woman who took a BP supplement [53]. This patient also suffered from seasonal allergic rhinitis only. Ingestion of BP also caused an anaphylactic reaction in a 56-year-old atopic woman, who was receiving allergen immunotherapy for allergic rhinitis and conjunctivitis [54]. In this case, anaphylaxis was not life-threatening. Another report by McNamara and Pien documented exercise-induced anaphylaxis associated with using bee pollen [55]. In this case, a 40-year-old male experienced a single episode of anaphylaxis while running outdoors. The patient had a history of seasonal allergic rhinitis, but had never previously experienced allergic symptoms from either bee pollen ingestion alone or exercise alone. He had been taking BP as a supplement for approximately one month before the event and had taken a dose of BP two hours before the run. There was also a paediatric case of exercise-augmented anaphylaxis [56]. A strong systemic allergic reaction was observed in a 15-year-old boy with a history of allergic rhinitis during exercise class. He never reacted to any other food or exercise. On the day of the event, he ate BP for breakfast. There is also a report from 1979 by Cohen et al. [57] describing three patients (25–31 years old) experiencing anaphylaxis after BP supplementation. Anaphylactic shock following BP ingestion was documented not only in adults/young adults, but also in a 4-year-old boy [58]. He was diagnosed with allergic rhinitis and had suffered from a fish allergy in early childhood.

The study by Pitsios et al. [59] tested the allergenic potential of BP in atopic patients (14–71 years old) with respiratory allergy to airborne pollen in comparison to a non-atopic control group (57 volunteers, age 14–70 years old). All participants underwent skin-prick testing with BP extracts. As a result of this study, strong cutaneous responses to BP were observed in atopic patients. These results indicate a high risk of allergic reactions to bee pollen consumption in sensitised individuals, highlighting the need for appropriate labelling of bee pollen-containing supplements and increased awareness among consumers with pollen allergies, even if they are not directly allergic to bee pollen.

The potential of BP to trigger skin reactions has also been confirmed through in vivo human studies. For instance, the research by Nonotte-Varly [60] performed skin-prick tests on 10 patients with a known allergy to *Oleaceae* pollen. The tests, which used three serial dilutions of ash (*Fraxinus*) bee pollen, demonstrated a clear-cut allergic reaction on the skin. The researchers found that the magnitude of the skin reaction, measured by wheal diameter, was directly proportional to the concentration of the bee pollen extract.

On the other hand, BP has demonstrated anti-allergic effects in both in vivo and in vitro studies [61,62]. In a murine model, an oral administration of BP significantly suppressed cutaneous mast cell activation induced by IgE and specific antigens. Complementary in vitro experiments revealed that BP reduced both mast cell degranulation and the production of tumour necrosis factor-α (TNF-α). This inhibitory action was attributed to BP’s ability to prevent IgE from binding to its receptor, FcεRI, on mast cells. Further evidence for this mechanism was provided by the observation of reduced protein tyrosine phosphorylation, a key downstream signalling event that occurs following FcεRI activation.

To date, no studies have assessed the in vitro or in vivo allergenicity of bee-collected pollen. However, research has focused on plant pollen, which is the primary constituent of bee pollen. For example, Gilles-Stein et al. [63] found that allergic skin reactions in humans are stronger when exposed to pollen allergens in the presence of low molecular weight compounds, which enhance the allergen-specific immune response in the skin and nose. Furthermore, in vitro studies have highlighted the crucial role of carbohydrates in modulating the immune response to plant pollen. For instance, Okano et al. [64] demonstrated that the carbohydrates on Cry j 1, the major allergen of *Cryptomeria japonica* (Japanese cedar), are essential for promoting a specific Th2 response; their removal significantly reduced T-cell proliferation and IL-5 production. Similarly, Batanero et al. [65] investigated Ole e 1 from *Olea europaea* (an olive tree) and found that the isolated carbohydrate component alone was capable of binding specific IgE and inducing histamine release from basophils. Collectively, these findings suggest that non-protein components, such as carbohydrates, are significant determinants of pollen allergenicity.

Allergies to ingested honeybee pollen occur far less frequently than allergies to airborne plant pollen. However, BP contains the same allergens; therefore, it can cause systemic reactions in sensitive people [49]. Since allergens identified in BP are classified into different plant families, the allergenic potential of this product depends strongly on the plant source, and thus, geographic origin, climate conditions, soil type, and even bee race [50]. BP can contain plant or fungal cross-reactive allergens [58,66]. In our previous study of BP proteins [67], we identified several allergens, including calmodulins, profilin, pollen-specific protein Bnm1, protein kinase homologue (Sal k 2), cobalamin-independent methionine synthase (Sal k 2), and cobalamin-independent methionine synthase (MetE). These allergens may be present in plants from different taxonomical families (including *Brassicaceae* and *Amaranthaceae*, among others). Profilin, along with other known allergens, such as prolamin, cystatin, expansin, Bras 2a, and alcohol dehydrogenase, was also identified in bee pollen derived from *Brassica campestris* (*Brassicaceae*) [68,69]. However, a study by Martín-Muñoz et al. [58] identified pollen from the *Asteraceae* family as a potential source of allergens. This family of plants, one of the largest in the world, is considered a major contributor to allergies [70]. It should be emphasised that, since bees collect pollen from a variety of plants (such as mugwort, goldenrod, sunflower, dandelion, and linden), which are known allergenic factors [71,72,73,74,75,76], a complete list of possible allergens present in bee pollen has not yet been identified.

### 3.3. Bee Bread

Bee bread (BB) is produced from bee pollen mixed with honey and bees’ digestive enzymes, which undergoes natural lactic fermentation within the hive [77]. Fermenting BP gives it many beneficial health properties. This product, like BP, possesses valuable health-promoting properties, such as antioxidant, antimicrobial, anti-inflammatory, hypotensive, anti-diabetic, hepatoprotective, immunomodulatory, anti-tumour, and anti-allergic effects [78,79,80]. Additionally, the presence of lactic acid bacteria may confer probiotic potential to bee bread. However, fermentation enriches the composition of BP, consequently enhancing its health benefits. During this process, anti-nutritional substances, such as phytates, oxalates, tannins, carbohydrates, and protein complexes are degraded through enzymatic hydrolysis, and the tough pollen exine is softened, making the minerals, vitamins (particularly B vitamins), amino acids, and proteins initially bound in non-absorbable complexes more digestible [81,82]. Additionally, microbial metabolism during fermentation transforms phytochemicals, particularly polyphenols, into more bioactive and bioavailable forms (e.g., by converting glycosides to aglycones), further enhancing the functional quality of the product [83]. Microorganisms, primarily lactic acid bacteria (e.g., *Lactobacillus* spp., *Streptococcus* spp., *Lactococcus* spp., etc.) and yeasts (e.g., *Saccharomyces* spp.), carry out the fermentation process and reduce the amount of toxic compounds through mechanisms such as enzymatic biodegradation and physical adsorption to microbial cell walls. These compounds include residues of antibiotics, pesticides, nitrates and aflatoxins produced by fungi [83,84]. It is worth noting that proteolytic enzymes active during lactic acid fermentation can hydrolyse allergenic proteins, potentially destroying IgE-binding epitopes and reducing the overall allergenicity of the product compared to raw bee pollen. However, the extent of this reduction varies and does not guarantee complete safety for highly sensitive individuals [85,86]. Bee pollen should be consumed in doses of up to 30 g per day for one month, followed by a break of several weeks between treatment cycles [87].

To date, there have been no reports of severe allergic reactions following the consumption of bee bread. It may be attributed to the lactic fermentation process in the hive, which can reduce the allergenicity of bee pollen. Regarding allergenicity, bee bread may therefore be considered a safer and more potent alternative to bee pollen.

The allergens present in BB are fundamentally the same as those found in bee pollen. However, the lactic acid fermentation process can hydrolyse allergenic proteins. This mechanism is thought to reduce the overall allergenicity of BB compared to raw bee pollen, which may explain why there have been no reports of severe allergic reactions following its consumption to date.

### 3.4. Royal Jelly

Royal jelly (RJ) is a nutritious, creamy substance secreted by the hypopharyngeal glands of worker bees, possessing numerous properties, such as anti-inflammatory, antioxidant, anti-diabetic, anti-ageing, anti-fatigue, anti-bacterial, anti-mutagenic, and antitumor [88,89] properties. For preventive purposes, a daily dose of 35–100 mg of RJ is recommended [90]. Higher doses are used for a more substantial therapeutic effect, typically ranging from 250 to 500 mg per day, and in some cases, up to 3 to 6 g daily [90,91,92]. Over a year, 2–3 treatment cycles lasting 6–8 weeks each should be carried out, preferably in the spring and autumn [90].

Although RJ supplementation is considered highly beneficial to health, it may cause severe allergic reactions in sensitive individuals. Anaphylaxis induced by RJ has been reported on several occasions. In Japan, a 26-year-old woman with a history of allergic rhinitis, bronchial asthma, allergic conjunctivitis, atopic dermatitis, and food allergy developed a severe allergic reaction after consuming a RJ and honey beverage [93]. One year earlier, also in Japan, a 33-year-old man experienced a severe systemic response following RJ ingestion [94]. Another case involved a 26-year-old Japanese woman with a history of bronchial asthma, but no previous allergies to HBPs, who was hospitalised for anaphylaxis after taking RJ tablets [95]. There was also a case of a 56-year-old woman who suffered from anaphylactic shock caused by RJ twice in one month [96]; it was the first documented RJ-induced anaphylactic case in China.

Several other cases of allergic reactions—including anaphylaxis and asthma—associated with RJ consumption have been reported in adults over the past century [97,98]. There is also evidence that RJ may cause contact dermatitis, as observed in a 30-year-old woman who used frozen RJ topically [99]. She had been using RJ for the treatment of her facial dermatitis. The evidence suggests that RJ poses an elevated risk to atopic populations. Specific IgE to RJ has been detected in as many as 17% of adult patients with asthma [100] and one third of patients with atopic dermatitis [101]. Therefore, individuals with a history of atopic diseases, such as atopic dermatitis AD, asthma, or allergic rhinitis, should avoid RJ products due to the potential risk of severe cross-reactive responses [101].

There is also one report documenting the development of haemorrhagic colitis, oedema, and infiltration of inflammatory cells in a 53-year-old woman with no history of cardiovascular diseases, after RJ supplementation for 25 days [102]. Moreover, the development of occupational asthma associated with royal jelly inhalation was reported [103]. A 43-year-old Chinese woman, working in the factory processing RJ for 11 years, developed wheezing while at work, which resolved after she left the factory. The patient was allergic to honeybee venom (HBV). In this case, cross-reactivity between RJ and HBV was confirmed.

There is also a report describing a severe anaphylactic reaction after the ingestion of RJ in combination with cefonicid, a cephalosporin antibiotic [104]. In this case, a 28-year-old man with a 25-year history of asthma developed severe dyspnea followed by loss of consciousness within 15 min after receiving a dose of cefonicid. The patient ingested royal jelly after the antibiotic was administered. In light of the described incident, particular care should be taken when taking medicines with unconventional food or supplements, as these can interact with each other and potentially lead to severe adverse effects.

There have also been reports of cases of anaphylaxis in children after consuming RJ. It should be emphasised that when introducing products that have the potential to cause allergies, such as RJ, to children, it is essential to exercise special care. An 11-year-old girl with a documented history of atopy died in 90s after ingestion of RJ [105]. Fortunately, this is the only documented case of a child dying after ingesting RJ. Nevertheless, milder reactions do occur, albeit rarely. For example, a 15-year-old atopic girl developed symptoms of anaphylaxis 15 min after the intake of RJ [106]. The reason for allergic reactions to RJ may be cross-reactivity between RJ and plant pollen. Although RJ is a pure secretion from bee glands, it may contain trace amounts of pollen collected by bees. An 11-year-old girl experienced a severe allergic reaction after drinking a beverage made of crude RJ [107]. In this child, a cross-reactivity between RJ and timothy hay (*Phleum pratense*) and ribwort plantain (*Plantago lanceolata*) was identified. An oral allergy syndrome after RJ intake also occurred in a 7-year-old girl who was diagnosed with a food allergy to almonds [108]. It is suspected that the allergic reaction was caused by RJ contamination with almond (*Prunus amygdalus*) pollen—a plant often placed near beehives.

Paradoxically, studies also describe the possible preventive effects of RJ against allergic reactions. Experiments in a murine model showed that allergy antigen-specific IgE production and histamine release from mast cells were suppressed following the administration of RJ to DNP-KLH mice. At the same time, macrophage function was restored and Th1/Th2 cell responses were improved [109]. Similar results were obtained in a study examining the preventive effects of RJ against anaphylactic shock associated with cow’s milk allergy in mice [110]. This study observed decreased serum IgG, IgE, and anti-β-Lg levels and plasma histamine levels. Thus, it may be concluded that RJ can potentially inhibit the degranulation of mast cells and, in some cases, can have a beneficial effect on the allergy, reducing its symptoms.

More recent studies have elucidated the specific molecular mechanisms driving this anti-allergenic potential, highlighting the role of 10-hydroxy-2-decenoic acid (10-HDA)—the major lipid constituent of RJ, and royalisin-derived peptides. For instance, Xu et al. [111] employed a combined approach, in which in vitro experiments on bone marrow-derived mast cells revealed that 10-HDA inhibits IgE-mediated activation by suppressing NF-κB (nuclear factor kappa B) signalling through the downregulation of histone H3 lactylation (H3K9la). These findings were corroborated in vivo, where 10-HDA alleviated allergic symptoms in mouse models of passive cutaneous and active systemic anaphylaxis. Furthermore, Sako et al. [112] focused on in vivo models, reporting that 10-HDA and biotinylated royalisin peptides alleviated anaphylactic symptoms in a rat hind-paw oedema model and a mouse model of anaphylactic hypothermia by targeting the platelet-activating factor (PAF), a critical mediator of allergic reactions. Thus, it may be concluded that RJ components can inhibit mast cell degranulation and potentially reduce allergic symptoms.

In addition to lipids and small peptides, specific proteins within RJ have also been identified as key immunomodulators. Okamoto et al. [113] isolated the Major Royal Jelly Protein 3 (MRJP3), a 70 kDa glycoprotein, and assessed its efficacy using both in vitro and in vivo approaches. In in vitro assays, MRJP3 significantly suppressed T-cell proliferation and the production of IL-4, IL-2, and IFN-γ (interferon-gamma). These findings were substantiated in vivo using an OVA/alum-immunised mouse model, where the intraperitoneal administration of MRJP3 inhibited serum levels of antigen-specific IgE and IgG1. Notably, the study revealed that heat-treated MRJP3 retained its ability to suppress allergic antibody responses while exhibiting reduced antigenicity compared to the native protein. This suggests that specific polypeptide regions within MRJP3 are responsible for its immunoregulatory activity, independent of its conformational antigenicity.

The leading cause of allergies after consuming RJ is the proteins from the Major Royal Jelly Protein family. In particular, Major Royal Jelly Proteins 1, 2, and 3 (MRJP1, MRJP2, and MRJP3, respectively) have been identified as the most potent allergens [95,96,114]. In our previous experimental study of RJ’s proteins and peptides [115], apart from MRJP8 and MRJP9, we identified venom acid phosphatase Acph-1-like, venom serine protease 34, and icarapin variant 2 precursor, which are the known allergens of HBV, and may cause severe reactions. As mentioned above, the allergic responses may also be attributed to the plant pollen grains in trace amounts in RJ [116]. Moreover, Hata et al. [101] identified six allergens that cross-react with RJ, namely the European house dust mite (HDM) (*Dermatophagoides pteronyssinus*), American HDM (*Dermatophagoides farinae*), snow crab (*Chionocetes* spp.), edible crab (*Cancer pagurus*), and German cockroach (*Blatella germanica*), as well as honeybee venom (*Apis mellifera*).

### 3.5. Propolis

Propolis, also called “bee glue”, is a natural, resinous mixture that is produced by honeybees from different exudates of plant sources, including branches, leaves, and buds [117]. Because of its strong bonding properties (hence the name “bee glue”), bees use it to construct and repair their hives—sealing holes in honeycombs or smoothing out the walls. Propolis is also used to protect the hive from external intruders [118,119]. It is generally seen as non-toxic as well as safe when consumed at around 70 mg a day [120]. Over 300 chemical substances have been identified in propolis [121]. It is high in benzoic acid, cinnamic alcohol, cinnamic acid, sesquiterpenes, triterpene hydrocarbons, heteroaromatic compounds, minerals, sterols, sugars, and amino acids [117]. Because of such a chemically dense profile, propolis has been linked to exhibit anti-bacterial, anti-fungal, anti-cancer, anti-viral, anti-inflammatory, and antioxidant properties [117,119]. Because of these beneficial bioactivities, propolis has become increasingly popular as a dietary supplement and is also contained in toothpastes, cosmetics, and ointments [122].

Several studies are addressing the allergenic potential of propolis. An 18-year-old female developed symptoms of burning pain in her lips 24 h after using propolis spray for gingival swelling [123]. On admission to the hospital, lip swelling and erythema around the mouth and under the right eye were also present. Her medical history involved epilepsy; however, patch testing proved that the allergic reaction was due to the propolis spray she used and not the medication she was taking. In another case, a 45-year-old beekeeper developed a psoriasiform contact dermatitis and believed it came from honeybee stings, which he was experiencing quite often due to the nature of his job [124]. However, upon evaluation by his doctors, it turned out that it was, in fact, a reaction to propolis, with which he had a lot of contact while collecting honey. There was also a serious case reporting a 59-year-old male with cholangiocarcinoma [125]. The patient was taking propolis for two weeks before he was admitted to the hospital with acute renal failure, which required hemodialysis.

However, despite these unarguably positive properties of propolis mentioned above, several allergens have been isolated from this product. These include caffeic acid esters—phenylethyl caffeic acid ester, 1,1-dimethylallyl caffeic acid ester (also called 3-methyl-2-butenyl caffeate), benzyl caffeate, geranyl caffeate, benzyl alcohol, benzyl cinnamate, methyl cinnamate, ferulic acid, and tectochrysin [120]. As seen in the two aforementioned cases [123,124], the most frequent symptoms after propolis ingestion included oedema, tongue and lip swelling, and oral pain. In contrast, the most common symptoms after non-oral exposure were eczema, erythema, and itching [35].

There are a couple of in vivo studies concerning propolis’ allergenicity. A Hungarian study [126] aimed to analyse data from 1992 to 2021, where over 17,000 pateints were patch tested. An increase in sensitization to propolis was seen in 2019–2021, which points at the recent increase in interest in natural products that was observed in recent years. The authors also point out cross-sensitivity with fragrance mix I (FMI) and balsam of Peru. Another study was conducted in 2023 in Italy and Sweden [127]. It showed that from 257 dermatitis patients in Italy, 6.2% reacted to propolis and 5.1% to propolis of Brazilian origin—showing that the origin of HBPs plays a role in sensitivity reactions.

It has been confirmed that propolis can induce both delayed-type (type IV) and immediate-type (type I) hypersensitivity reactions [128]. Consequently, the topical application of propolis carries a risk of cross-reactivity with products containing structurally similar components. Specifically, cross-allergic reactions have been documented between propolis and Peru balsam, rosin, turpentine, essential oils, and various fragrances. However, despite this overlap, the literature indicates that the incidence of hypersensitivity reactions to propolis is approximately two to three times lower than that of Peru balsam [128].

### 3.6. Beeswax

Beeswax (BW) is a naturally occurring, complex mixture secreted from the wax glands of worker bees [129,130]. It is made up of over 300 constituents, including hydrocarbons, free fatty alcohols, esters of fatty acids and fatty alcohols, diesters, and exogenous substances. The latter refers to the trace amounts of propolis and pollen, as well as some pollutants [131]. BW can be divided into two distinct types: yellow BW and white BW, obtained through bleaching and purification of yellow beeswax [132]. In the European Union, it is used as a food additive under the symbol E 901 [133,134]. Other than that, it is also broadly used in the skincare industry, and thanks to its chemical composition, it can act either as an occlusive, a humectant, or an emollient [129].

Allergic reactions to BW are relatively rare compared to its widespread use, especially in the cosmetic industry. If they happen, they result more from propolis contamination—especially caffeic acid derivatives [129,132]. There is a case of a 26-year-old dental technician who developed pulpitis and fingertip and nail eczema in his right thumb and index finger [135]. He had a history of atopic eczema. At work, he was moulding BW between his index finger and thumb in order to make a dental crown. Patch testing revealed a positive reaction to propolis.

Rajpara et al. conducted an in vivo study that assessed both the prevalence and clinical relevance of positive patch tests to propolis and evaluated its cross-reactivity with BW, colophonium, FMI, and *Myroxylon pereirae* [136]. The results showed that 55 patients out of 2828 were allergic to propolis, which was 1.9%. Then, the allergy to BW was equal to 0.45%, which was 13 patients out of 2828 tested. Cross-reactivity of BW to propolis was seen in 4 out of 55 patients, which makes it 7%. While the numbers for BW are not very high, it should be taken into account that allergic reactions can happen, especially in people already sensitised to propolis.

In another in vivo study, by Nyman et al. [132], patients with cheilitis or facial dermatitis were tested for contact allergy to BW and propolis. Ninety-five patients took part in the study (age range 18–83 years); 41 patients had a history of atopic eczema, and for 15, there was no such information. Seventeen patients reacted positively to BW; out of those 17, 14 were tested with yellow and white BW. The result was that eight were positive for both types of BW, 5 for yellow BW, and just 1 for white BW. As the authors note, the prevalence of BW allergy seems relatively high in this study due to the enrolled population of patients. Nonetheless, it should be pointed out that even though BW allergy can still be considered rare, atopic patients should be patch tested for both propolis and BW.

In terms of in vitro studies, Mekky et al. [137] used African green money kidney cells (also called Vero cells) to assess the cytotoxicity of BW and found that it does, in fact, toxically affect cells. For assessment of the proportion of viable cells, the MTT (3-(4,5-dimethylthiazol-2-yl)-2,5-diphenyltetrazolium bromide) test was used, and it showed that BW’s toxicity is concentration-dependent.

There is a lack of information about the allergens present in BW. What is established is that the allergic reactions that patients experience are mostly due to BW’s contamination with propolis. However, as in the study mentioned above, reactions to white BW can happen, even though it is a purified version of yellow BW, indicating that there might be something else causing the reaction—it might be an area for further research to compare chemical composition of yellow and white BW and their impact on the prevalence of allergy.

### 3.7. Bee Brood

Bee brood refers to three distinct stages (eggs, larvae, and pupae) of honeybee development before they become adults. While less common as a supplement than other beehive products, bee brood is gaining popularity as an alternative food source. Already widely consumed in regions like Asia, Central America, and Australia, it is now under consideration for approval as a “novel food” in Europe [138,139]. Bee brood is recognised as a highly nutritious food source. Its composition includes macronutrients, such as proteins, carbohydrates, and lipids. It is considered a complete protein source, providing all essential amino acids. Moreover, it is a valuable source of B-vitamins, vitamin C, and choline, complemented by a wide array of essential macro- and micronutrients such as P, Mg, K, Fe, Zn, Co, and Se [140,141].

The allergenic potential of drone larvae remains poorly understood. There is only one report published in 2018 documenting anaphylaxis developed in a 29-year-old male after drinking a beverage composed of raw drone larvae for the first time [142]. The patient had a history of asthma and mild allergic rhinitis. He was stung by bees several times before, but never experienced a severe allergic reaction. He also tolerated honey, royal jelly, and propolis well.

So far, the allergenicity of bee brood has not been assessed using in vitro or in vivo models. However, significant insights can be drawn from reports assessing the safety of other edible insects. For instance, Nicoletta et al. [143] investigated the Black Soldier Fly (*Hermetia illucens*) and observed a high rate of in vitro IgE cross-reactivity to recombinant tropomyosin and arginine kinase in subjects already sensitised to crustaceans and mites. These findings confirm that conserved pan-allergens pose a significant risk to atopic individuals. Similar risks have been identified in studies on the yellow mealworm (*Tenebrio molitor*). Bajuk et al. [144] utilised a canine model to investigate the interaction between mealworm proteins and the immune system of dogs sensitised to storage mites. Western blot analysis confirmed that IgE from allergic dogs bound specifically to mealworm proteins, and mass spectrometry identified 17 proteins, including tropomyosin and α-amylase, as cross-reacting allergens. This suggests that the risk of cross-reactivity is not limited to human models but is a biological property of these conserved arthropod proteins.

Although allergic reactions following bee brood consumption are rare (or poorly documented), several allergens in this product were identified. In our previous study on the proteomic composition of honeybee larvae [138], we identified several proteins that may be considered allergens, including arginine kinase, glyceraldehyde-3-phosphate dehydrogenase, thioredoxin, MRJP1, MRJP2, and alpha-glucosidase. However, studies of the allergenicity of bee brood are very scarce. The only research conducted in Russia reports an incidence of allergy to drone brood application in 2.4% of the tested population (*n* = 41) [145]. Brood may contain honey, RJ, propolis, wax, and nectar. Thus, consuming bee brood may cause mild to severe symptoms in patients with allergies [139].

A summary of the allergenic profiles, immunological mechanisms, and cross-reactivity of honeybee products is provided in Table 1.

### 3.8. Toxicological Assessment of HBPs via In Vitro Models

Beyond allergenicity, the general cytotoxicity of bee products requires rigorous assessment to ensure consumer safety, particularly in relation to topical applications. In vitro models offer a valuable complement to standard toxicological studies. The HaCaT cell line (immortalised human keratinocytes) is widely recognised as a robust model for assessing the cutaneous toxicity of HBPs used in dermatology. Recent studies on linden honeydew honey and various Indian honey varieties incorporated into hydrogels have demonstrated that these products exhibit no cytotoxic effects on HaCaT cells at therapeutic concentrations, instead promoting wound closure and tissue repair [146,147]. However, researchers must exercise caution when selecting assays. Abel and Baird [148] reported that honey’s reducing sugars and enzymes can interact with reagents in standard MTT and LDH (lactate dehydrogenase) assays, potentially generating false-negative toxicity results. Therefore, alternative methods, such as the sulforhodamine B (SRB) assay or dual staining (Propidium Iodide/Hoechst), are recommended for accurate safety profiling.

Toxicological profiles also vary significantly by bee species and product type. In a comparative study of Egyptian bee products, Badria et al. [149] found that bee venom, expectedly, exhibited the highest cytotoxicity against liver and breast cancer lines, followed by propolis, while honey showed minimal activity. Salazar-Olivo and Paz-González [150] demonstrated that RJ protein fractions could stimulate growth in insect cells while exhibiting cytotoxic effects on human cervical carcinoma (HeLa) cells, suggesting a complex, context-dependent bioactivity. Research on stingless bee (*Trigona* spp. and *Melipona* spp.) products, including honey, propolis, and bee pollen, has revealed significant cytotoxic activity against various human cancer cell lines, such as lung adenocarcinoma (A549), liver (HepG2), and cervical (HeLa) cancer cells [151,152,153,154].

Furthermore, the potential of RJ and BB as selective anti-cancer agents is gaining attention. Recent studies have shown that RJ exerts selective cytotoxicity against lung malignant cells (A549) [155] and human hepatoma cells (HepG2) [156] via apoptotic pathways, while showing no cytotoxicity toward normal human cells (MRC-5 and THLE-3). Ulusal et al. [157] confirmed similar apoptotic effects in acute myeloid leukaemia (HL-60) cells. Bee bread has also demonstrated moderate cytotoxicity against lung, prostate, and neuroblastoma cell lines [158].

Crucially, selectivity between cancer cells and healthy cells must be established. For instance, Bonamigo et al. [159] reported that Brazilian propolis extracts (from *P. droryana* and *A. mellifera*) induced necrosis in erythroleukemia cells while showing significantly lower cytotoxicity toward healthy peripheral blood mononuclear cells (PBMCs). Conversely, Teerasripreecha et al. [160] isolated cardanol and cardol compounds from Thai propolis that were cytotoxic not only to cancer lines but also to healthy human fibroblasts (Hs27). These findings underscore the need for future safety protocols to integrate in vitro cytotoxicity assays using non-tumorigenic models (e.g., Hs27, HaCaT, Caco-2) to ensure that HBP supplementation does not pose risks to healthy tissue.

## 4. Diagnosis and Management of HBP Allergy

Given the diverse nature of honeybee products, the clinical presentation and management of allergic reactions can vary significantly. Table 2 summarises the frequency and severity of these reactions, ranging from mild contact dermatitis to systemic anaphylaxis. Furthermore, it identifies specific high-risk populations, such as individuals with asthma or those exposed to occupational hazards, and correlates them with the appropriate diagnostic methods and therapeutic interventions described in the subsections below.

### 4.1. Diagnostic Approaches to HBP Allergy

Bee venom allergy is the most commonly diagnosed allergy related to bees. When evaluating a patient for HBP allergy, it is essential to distinguish it from *Hymenoptera* venom allergy, as the allergens and clinical management differ.

Accurate diagnosis of HBP allergy is challenging due to the complex composition of these products and the high prevalence of cross-reactivity. Diagnostic protocols often mirror those used for venom allergy, relying on a combination of in vivo and in vitro methods [161].

The diagnostic protocol for IgE-mediated food allergy, including HBP allergy, is based on a detailed clinical history and usually begins with skin-prick tests (SPTs) and the measurement of sIgE levels in blood [161,162]. SPTs remain the first-line diagnostic tool. However, a critical limitation in HBP allergy is the lack of standardised commercial extracts compared to the available standardised honeybee venom extracts. Most case reports rely on “prick-to-prick” tests using the raw product involved in the reaction [36,108,142,163]. While effective, this introduces variability in allergen concentration and stability. Furthermore, as observed in venom diagnostics, tests using whole extracts may result in frequent double sensitisations or, conversely, false negatives if the extract contains too little of a specific allergenic component (e.g., Api m 10). Therefore, it is crucial to develop standardised extracts of HBPs suitable for accurate skin-prick testing.

Measurement of sIgE in human serum is considered a standard, yet interpretation is complicated by cross-reactive carbohydrate determinants (CCDs) [164]. Many allergens are glycoproteins with N-glycan structures containing core α1,3-fucose and β1,2-xylose. IgE antibodies may bind to these carbohydrate moieties rather than the protein peptide [165,166,167]. As shown in venom allergy studies, up to 75% of double sensitisations are due to CCDs, often resulting in clinically irrelevant false positives [161].

To overcome the limitations of whole-extract testing, component-resolved diagnostics (CRD) utilising recombinant allergens without naturally occurring insect glycosylation (CCD-free) is essential. This method allows for the differentiation between genuine sensitisation and cross-reactivity (e.g., distinguishing true allergy from reactions caused by pollen-food syndrome) [161]. In the context of bee venom, using a panel of recombinant allergens (e.g., rApi m 1, rApi m 10) significantly increases diagnostic sensitivity; applying this approach to HBP diagnosis is crucial to prevent unnecessary dietary restrictions.

For equivocal cases, the basophil activation test, BAT, offers a functional assay that mimics the in vivo allergic response in a test tube [161]. Recent studies suggest that BAT, although possibly less sensitive and specific compared to sIgE testing, may serve as a crucial tool when skin testing is contraindicated.

Allergy to honey and BP is mainly diagnosed through serological sIgE in vitro measurement and skin-prick tests [52,168]. Allergy to RJ is primarily diagnosed based on the results of the skin-prick tests [8,94,95], as well as sIgE testing and BAT [95,96]. Allergy to propolis may be diagnosed using a patch test [127]. In the study by Nyman et al. [132] and Rajpara et al. [136], patch tests were also used to assess allergies to propolis and beeswax. The second study stated that the patch tests were administered with Finn chambers, which is a system consisting of ten aluminium chambers attached to a panel made from nonocclusive tape with an acrylic-based adhesive, which is hypoallergenic [169]. If the diagnosis of the allergy to specific HBPs remains uncertain, an oral food challenge under medical supervision may be necessary to confirm clinical reactivity. Additionally, CRD may help to identify the specific allergens present in HBPs, thereby improving diagnostic accuracy and medical management of HBP allergy [170]. Cross-reactivity with other pollens or bee products should also be considered during diagnosis.

### 4.2. Pharmacological Management of HBP Allergy

Management of HBP allergy relies primarily on strict avoidance of these products, but understanding the mechanisms of rescue medication is vital for acute treatment. Severe, life-threatening anaphylaxis caused by HBPs is treated in hospitals using epinephrine, antihistamines, glucocorticosteroids, and bronchodilators [31,52,94]. Epinephrine (adrenaline) is the only first-line treatment for HBP-induced anaphylaxis. Its mechanism involves agonism of α-adrenergic receptors (inducing vasoconstriction to reverse hypotension and reduce mucosal oedema) and β-adrenergic receptors (causing bronchodilation and inhibiting further mediator release from mast cells/basophils) [171]. Because HBP reactions can be rapid and systemic, delayed administration of epinephrine is a major risk factor for mortality. Antihistamines (H1-antagonists) are used as adjunctive medications to prevent allergy symptoms. Examples of these drugs include cetirizine or diphenhydramine. They block the H1 receptors on target tissues [172]. While they alleviate cutaneous symptoms (urticaria and itching), they do not stabilise mast cells or prevent life-threatening respiratory or cardiovascular compromise. They are considered third-line interventions, administered after adrenaline (first-line intervention) and inhaled short-acting beta-2 agonists (second-line interventions) [173]. Glucocorticosteroids, also utilised in the management of HBP allergy, reduce the synthesis of inflammatory cytokines (leukotrienes and prostaglandins) and may prevent the biphasic (late-phase) anaphylactic response [174]. However, their onset of action is slow (4–6 h), making them ineffective for acute symptom arrest [175]. Moreover, given the risk of adverse events and the absence of robust evidence supporting their efficacy in mitigating severity or preventing biphasic reactions, it is not recommended to administer the corticosteroids in anaphylaxis [176].

After experiencing a severe allergic reaction related to HBPs, patients are typically advised to discontinue use of the offending product and exercise caution with other HBPs. They may also be prescribed an epinephrine auto-injector for emergency use in case of a recurrent reaction [8,55]. It is vital for allergic people who must avoid beehive products to carefully check the labels of dietary supplements and cosmetics for the presence of these ingredients. Mandatory labelling of potential allergens on consumer packaging of products containing HBPs, especially bee pollen and honey, has been recommended by several studies [58,93,177]. This is particularly important because honey and other bee products are often hidden ingredients in processed foods such as energy bars, cereals, candies, cakes, and biscuits, potentially exposing sensitive individuals to allergens unknowingly [35].

### 4.3. Future Directions in Research and Clinical Management

Despite the growing consumption of bee products, significant gaps remain in ensuring patient safety. Future research must prioritise the development of standardised, commercial diagnostic extracts to replace the highly variable “prick-to-prick” method. Furthermore, extensive molecular characterisation of HBP allergens is required to expand component-resolved diagnostics (CRD) panels; this will allow clinicians to better distinguish between genuine HBP allergy and pollen-food cross-reactivity. Finally, clinical trials are needed to establish safe protocols for oral immunotherapy (OIT) or desensitisation specifically for beekeepers and frequent consumers of apitherapy products who develop severe allergies.

## 5. At-Risk Populations and Confounding Factors

Although allergies to HBPs, including life-threatening anaphylactic reactions, are relatively rare, some groups of people are more vulnerable and should be cautious when using HBPs, especially for the first time. According to published case reports, people with a history of asthma, eczema, or allergic rhinitis (hay fever) are at the highest risk of developing anaphylaxis after taking HBPs [32,52,53,54,55,56,57,93,105,135]. However, there are also documented cases of people who developed severe allergic reactions after consuming honey, even though they had no previous history of atopy [33]. Therefore, it should be emphasised that even in people without known allergies, HBPs should be used cautiously, starting with small doses. Caution should also be exercised when administering HBPs to children. Specifically, due to the risk of infant botulism from *Clostridium botulinum* spores, honey should not be given to children under 12 months of age [178]. On the other hand, the introduction of honey should not be delayed for too long. Delayed introduction of diverse foods into a child’s diet may paradoxically increase the risk of allergic sensitisation [178]. Therefore, after the first year of life, honey can generally be introduced as part of a balanced diet. It is also worth noting that some mild adverse events may occasionally occur in children after honey consumption, including hyperactivity, nervousness, and insomnia [179].

Another potential concern relates to environmental contamination. A global survey demonstrated that a majority of analysed honey samples contained quantifiable amounts of neonicotinoids, which are among the most widely used classes of insecticides [180]. However, in all cases analysed, the concentrations of these contaminants were below the established maximum authorised limits for human consumption as defined by European Union and U.S. regulations. Consequently, honey consumption is generally not considered a health risk to humans based on current regulatory standards.

Regarding other bee products, the primary concern is the potential for allergic reactions, as well as the lack of specific safety data in paediatric populations. Propolis is a known contact sensitiser, and its use, particularly topical, in children is often discouraged due to the risk of allergic contact dermatitis. However, in a study by Kara et al. [181], no adverse effects were observed after propolis administration to 87 children with hand, foot, and mouth disease. Royal jelly contains hormonally active compounds [182], and its effect on developing children is not well-established, warranting caution or avoidance in young children unless medically supervised. On the other hand, a study of Zahran et al. [183] has proven the positive effects of RJ in treating systemic lupus erythematosus in children. RJ is also recommended for supplementation to treat other diseases in children, including a weak immune system, impairment of the nervous system, weakness, loss of appetite, anorexia, and anaemia [184].

Another specific group with elevated risk of developing adverse reactions are pregnant women. In general, honey is recommended during pregnancy to treat various conditions, such as sore throat, cough, and urinary tract infections, primarily due to its anti-bacterial properties [185,186]. Moreover, honey can support the treatment of anaemia during pregnancy [187,188,189]. Propolis is also considered relatively safe for pregnant women and may be useful for treating bacterial diseases, such as bacterial vaginosis [190]. The research conducted in vivo on rats and mice suggests that propolis can be safely used to improve pregnancy outcomes and reduce placental oxidative stress [191,192,193]. Similarly, a study conducted on rats suggests that another HBP, bee bread, may have beneficial effects on the adverse effects of pregnancy [187]. Other bee products are also considered generally safe during pregnancy [194]. However, it has been suggested that pregnant women should avoid royal jelly due to their potential hormonal activity or lack of clear safety data, particularly in high doses [195,196].

Beekeepers are also particularly vulnerable to developing an allergy to HBPs. While their primary risk is venom stings, they can develop contact dermatitis from handling other HBPs, especially propolis. A 2012 study on propolis allergy among Polish beekeepers [197] found that, although not common, beekeepers are more susceptible to propolis allergy than the general population. This study, which was based on a questionnaire, reported a propolis allergy rate of 3.05%, corresponding to 17 out of 558 beekeepers. Based on the answers from the questionnaire, 2 out of those 17 beekeepers reported an extreme reaction to propolis, six experienced a moderate reaction, and nine had a slight response. In another study conducted in India among beekeepers [198], patch testing with self-prepared propolis antigens revealed that propolis acted as a primary sensitiser in this group. Moreover, based on a survey by Münstedt et al., contact allergy to propolis is common among beekeepers [122]. However, beekeepers are usually unaware of their condition and continue to work with HBPs without protective equipment such as gloves. They even use propolis as a treatment for other diseases. Münstedt et al. [122] stated that allergies were more commonly experienced by beekeepers who had stronger reactions to bee stings in the spring, after a period without stings, and those with benign lung disease, those who experienced other allergies, and those who spent more time playing sports. They also reported that propolis allergies in beekeepers were unrelated to gender, age, body mass index, years of beekeeping, reactions to bee stings, or the number of hives. Since there is currently no effective treatment for propolis contact allergy, heat immersion therapy has been proposed as a potential relief for symptoms; however, this approach requires further testing and validation [199].

It should also be kept in mind that bee products are highly heterogeneous and that their composition varies depending on the geography of their origin, climate, season, and the presence of specific honey plants [200]. Therefore, HBPs produced in different countries may have varying allergenic potential, which should be considered when assessing the allergenicity of HBPs in sensitive individuals.

## 6. Gaps in Knowledge and Future Directions

Although case studies have demonstrated the allergenic potential of bee products, the precise characteristics of their bioactive components remain unclear [67,115,138]. This is primarily due to the significant variability of bee products depending on their geographic origin, as well as the complexity and challenges in identifying all their constituent compounds [201]. The complexity and geographical variability of bee products pose a significant challenge to standardisation and safety assessment. Moreover, the allergenicity of bee products varies from person to person and often appears to have a great deal of randomness. While the vast diversity of, e.g., honey types and pollen sources makes it difficult to attribute allergenicity to a single substance universally, identifying conserved allergenic proteins (such as Major Royal Jelly Proteins) versus variable environmental contaminants is crucial. Therefore, research into the components contained in bee products and their bioactivity should continue, using state-of-the-art analytical tools such as mass spectrometry (MS), high-performance liquid chromatography (HPLC), gas chromatography (GC) methods, and nuclear magnetic resonance (NMR) spectroscopy [202,203]. In addition, in vitro and in silico analyses should be conducted to investigate the biological activity of known and previously unidentified components of bee products. A deeper understanding of these components and their bioactivity would help clarify the mechanisms underlying HBP allergies and support the development of improved management strategies. Also, it would be beneficial to develop standardised allergen extracts to facilitate the proper diagnosis of HBP allergy and for research purposes. Future studies should also investigate potential cross-reactivity between allergens included in HBPs and homologous proteins in other insects or foods. This topic is vital in the context of the proper and accurate diagnosis of food allergy and the interpretation of sensitisation profiles. By identifying the potential cross-reactivity, sensitive individuals could be aware of the potential anaphylaxis risk and avoid consuming HBPs.

Moreover, prospective studies should be conducted to determine the prevalence and incidence of HBP allergies and assess the actual risk of developing this condition among different populations (e.g., adults, children, and beekeepers).

## 7. Conclusions

Honeybee products, including honey, bee pollen, royal jelly, propolis, beeswax, and bee brood, are increasingly popular for their health benefits, primarily due to growing consumer awareness of healthy lifestyles and a preference for natural products. However, HBPs are an under-recognised source of allergens and pose a significant risk of allergic reactions, including rare but potentially life-threatening anaphylaxis. High-risk groups, including people with atopic conditions and beekeepers, should use HBPs with special caution. Diagnosing these adverse allergic reactions, which can be either Type I (IgE-mediated) immediate hypersensitivity (e.g., anaphylaxis from honey or royal jelly) or Type IV (T-cell-mediated) delayed hypersensitivity (e.g., contact dermatitis from propolis), requires a tailored approach starting with clinical history, skin-prick tests (SPT), and specific IgE (sIgE) measurement. Diagnostic challenges include the lack of standardised commercial extracts for SPTs, often necessitating the variable “prick-to-prick” method, and the interference of cross-reactive carbohydrate determinants (CCDs) with sIgE testing, which can lead to false positives. To improve diagnostic accuracy and distinguish genuine allergy from cross-reactivity, such as Pollen-Food Allergy Syndrome (PFAS), Component-Resolved Diagnostics (CRD) utilising recombinant, CCD-free allergens is becoming crucial. For severe allergic reactions, management involves strict avoidance of the HBP, and for anaphylaxis, immediate treatment with epinephrine (adrenaline), the only first-line medication that rapidly acts to reverse cardiovascular and respiratory compromise via α- and β-adrenergic receptor agonism. Adjunctive therapies include antihistamines for cutaneous symptoms, but they are not effective for severe systemic reactions. Recommendations to improve safety include mandatory and precise product labelling, conducting a small-dose trial or SPT before use, and providing sensitised individuals with an epinephrine auto-injector. Further research is essential to clarify the underlying allergenic mechanisms and enhance safety protocols for all users.

## Figures and Tables

**Table 1:** Summary of allergenic profiles, immunological mechanisms, and cross-reactivity of honeybee products (HBPs).

Bee Product	Primary Mechanism	Main Clinical Manifestation	Known/Suspected Allergens	Notes on Cross-Reactivity
Honey	Type I (IgE-mediated)	Anaphylaxis, urticaria, angioedema, gastrointestinal symptoms	Bee origin: Major Royal Jelly Protein 1 (MRJP1) Plant origin: Pollen proteins (e.g., from *Asteraceae* or *Compositae* families)	Strong cross-reactivity with airborne pollens (Pollen-Food Allergy Syndrome)
Bee Pollen	Type I (IgE-mediated)	Anaphylaxis	Pan-allergens: calmodulins, profilin, Specifics: Bnm1, Sal k 2, MetE	Highly dependent on botanical origin. Frequent cross-reactivity with plant pollens (weeds, grasses, trees)
Bee Bread	Type I (IgE-mediated)	-	Similar profile to BP, but potentially modified by fermentation	Lactic acid fermentation may degrade some allergenic epitopes, potentially lowering allergenicity compared to raw pollen
Royal Jelly	Type I (IgE-mediated)	Anaphylaxis, asthma, contact dermatitis	Major Royal Jelly Proteins, in particular: MRJP1, MRJP2, MRJP3 Others: venom acid phosphatase Acph-1-like, venom serine protease 34, icarapin variant 2 precursor	Cross-reactivity observed with honeybee venom and environmental allergens (dust mites, crab, cockroach)
Propolis	Type IV (T-cell-mediated) Type I (IgE-mediated)	Allergic contact dermatitis	Caffeates: 3-methyl-2-butenyl caffeate, phenylethyl caffeate, geranyl caffeate, benzyl caffeate, Others: Ferulic acid, tectochrysin, benzyl cinnamate, methyl cinnamate	Cross-reactivity with Peru balsam, rosin, turpentine, essential oils, and various fragrances
Beeswax	Type IV (T-cell-mediated)	Contact dermatitis	Contaminants: mainly caffeic acid derivatives	Pure beeswax is generally inert; reactions are usually due to propolis impurities remaining in unrefined wax
Bee Brood	Type I (IgE-mediated)	Anaphylaxis	Bee origin: MRJP Pan-allergens: arginine kinase, glyceraldehyde-3-phosphate dehydrogenase, thioredoxin, and alpha-glucosidase	Risk of cross-reactivity with other honeybee products

**Table 2:** Clinical overview of honeybee product allergies.

Product	Frequency and Risk	Severity and Clinical Picture	High-Risk Populations	Diagnostic Approach	Management
Honey	Rare incidence, though widely consumed	Mild to severe; ranges from oral itching to systemic anaphylaxis and collapse	Individuals with atopy, specifically pollen allergies (*Asteraceae*) or food allergies	sIgE measurement, skin-prick test (SPT) (often prick-to-prick), component-resolved diagnostics (CRD)	Strict avoidance, epinephrine (adrenaline) for anaphylaxis; antihistamines for mild cutaneous symptoms
Bee Pollen	Uncommon, but the risk increases with atopy	Severe; high risk of anaphylaxis, bronchoconstriction, and angioedema within minutes of ingestion	Patients with seasonal allergic rhinitis (hay fever) and pollen hypersensitivity	SPT with pollen extracts, sIgE	Discontinuation of supplements, emergency epinephrine auto-injector for sensitised individuals
Bee Bread	Very rare, considered safer than raw bee pollen	Mild; no anaphylactic reactions reported to date; mainly mild gastrointestinal or cutaneous symptoms	Individuals sensitive to raw bee pollen	Similarly to bee pollen; oral food challenge may be considered under supervision	Avoidance if pollen allergy is confirmed
Royal Jelly	Rare, but may be dangerous	Severe; history of fatal and near-fatal anaphylaxis; acute asthma attacks	Asthmatics (highest risk), individuals with atopic dermatitis or honeybee venom allergy	SPT, sIgE, basophil activation test (BAT)	Strict avoidance for asthmatics/atopics; epinephrine for acute reactions
Propolis	Common; related to occupational risk	Mild to moderate; primarily delayed contact dermatitis (eczema, swelling); systemic reactions are rare	Beekeepers (occupational exposure), users of natural cosmetics and biocides	Patch testing (gold standard for type IV hypersensitivity)	Topical corticosteroids for dermatitis; avoidance of propolis-containing cosmetics/lozenges
Beeswax	Rare, often linked to impurity	Mild; localised contact dermatitis, cheilitis, or fingertip eczema	Individuals already sensitised to propolis or fragrances	Patch testing (differentiating between yellow and white wax)	Avoidance of unrefined wax products; use of purified synthetic alternatives
Bee Brood	Rare—emerging novel food	Moderate; potential for anaphylaxis due to pan-allergens	Individuals allergic to crustaceans (shellfish), dust mites, or mealworms	sIgE (potential cross-reactivity)	Avoidance of edible insects; epinephrine for accidental ingestion reactions

## Data Availability

No new data were created or analyzed in this study. Data sharing is not applicable to this article.

## Abbreviations

The following abbreviations are used in this manuscript:

10-HDA	10-hydroxy-2-decenoic acid
APC	Antigen-presenting cells
BAS	Basophils
BAT	Basophil activation test
BB	Bee bread
BP	Bee pollen
BPH	Birch pollen honey
BW	Beeswax
CCDs	Cross-reactive carbohydrate determinants
CD40L	Cluster of Differentiation 40 Ligand
CRD	Component-resolved diagnostics
DC	Dendritic cells
FMI	Fragrance mix I
GC	Gas chromatography
HBPs	Honeybee products
HBV	Honeybee venom
HDM	House dust mite
HPLC	High-performance liquid chromatography
ICU	Intensive care unit
IFN-γ	Interferon-gamma
IgE	Immunoglobulin E
IL	Interleukin
ILC2	Innate lymphoid type 2 cells
LDH	Lactate dehydrogenase
Mφ	Macrophages
MC	Mast cells
MetE	Cobalamin-independent methionine synthase
MHC II	Major histocompatibility complex class II
MMP-9	Matrix metalloproteinase 9
MS	Mass spectrometry
MRJP	Major royal jelly protein
MTT	3-(4,5-dimethylthiazol-2-yl)-2,5-diphenyltetrazolium bromide test
NF-κB	Nuclear factor kappa B
NK-T	Natural Killer T cells
NMR	Nuclear magnetic resonance
OIT	Oral immunotherapy
PAF	Platelet-activating factor
PFAS	Pollen-Food Allergy Syndrome
RAST	Radioallergosorbent test
RJ	Royal jelly
RH	Regular honey
sIgE	Specific Immunoglobulin E
SPT	Skin-prick test
SRB	Sulforhodamine B
Tfh	T follicular helper
Th	Naïve T-helper cells
TNF-α	Tumour necrosis factor alpha
TNF-β	Tumour necrosis factor beta
Treg	Regulatory T cells
TSLP	Thymic stromal lymphopoietin
